# Fruit and vegetable consumption and emotional distress tolerance as potential links between food insecurity and poor physical and mental health among homeless adults

**DOI:** 10.1016/j.pmedr.2019.100824

**Published:** 2019-02-08

**Authors:** Daphne C. Hernandez, Sajeevika S. Daundasekara, Katherine R. Arlinghaus, Anika Pal Sharma, Lorraine R. Reitzel, Darla E. Kendzor, Michael S. Businelle

**Affiliations:** a The University of Houston, Department of Health, & Health Performance, 3875 Holman Street, Garrison Gymnasium, Room 104, Houston, TX 77204-6015, USA; b The University of Houston, HEALTH Research Institute, 4849 Calhoun Road, Houston, TX 77204, USA; c The University of Houston, Honors College, 4333 University Drive, Houston, TX 77204-2001, USA; d The University of Houston, Department of Psychological, Health, & Learning Sciences, Social Determinants/Health Disparities Lab, 3657 Cullen Blvd Stephen Power Farish Hall, Houston, TX 77204-5029, USA; e The University of Oklahoma Health Sciences Center, Department of Family and Preventive Medicine, USA; f The University of Oklahoma Health Sciences Center, Stephenson Cancer Center, Oklahoma Tobacco Research Center, 655 Research Parkway, Suite 400, Oklahoma City, OK 73104, USA

## Abstract

Food insecurity is associated with mental health outcomes among adults experiencing homelessness. Different theoretical explanations have emerged to account for the negative health outcomes among vulnerable populations. The neomaterial theoretical perspective suggests that nutritional deficiencies from experiencing food insecurity are related to negative health outcomes. Whereas, the psychosocial theoretical perspective indicates that perceived disadvantages or inability to cope emotionally (i.e. lower distress tolerance) from food insecurity leads to adverse health outcomes. Building on these theoretical perspectives, the purpose of the study was to determine whether fruit and vegetable consumption (as a measure of diet quality) or emotional distress tolerance act as potential links between food insecurity and poor physical and mental health among adults experiencing homelessness. Adults were recruited from six area shelters in Oklahoma City (N = 566) during July–August 2016. Data was collected via a self-administered questionnaire on a tablet computer. Self-rated poor health, depression, and post-traumatic stress disorder (PTSD) were regressed on food insecurity using logistic regressions. Indirect effects were assessed using bootstrapping methods outlined by Preacher and Hayes. In covariate-adjusted models, lower levels of distress tolerance, but not fruit and vegetable consumption, partially mediated the association between food insecurity and poor health (β = 0.28, [0.14, 0.44]), depression (β = 0.56, [0.33, 0.88]), and PTSD (β = 0.39, [0.22, 0.60]). Results suggest that experiencing food insecurity may lower the ability to withstand emotional distress and consequently contributes to negative health outcomes.

## Highlights

• Among homeless adults, a positive link exists between food insecurity and poor health.
• Distress tolerance partially mediates the relation between food insecurity and health.
• Fruit and vegetable intake is not a mediator among homeless adults.

## 1 Introduction

Food insecurity is a major public health concern. This represents 15.6 million households without consistent access to adequate food due to lack of money or other resources at times during the year (Coleman-Jensen et al., 2017). In 2016, 12.3% of American households (41.2 million people) were food insecure (Coleman-Jensen et al., 2017). However, the numbers reported by USDA are based on domiciled samples and national numbers of non-domiciled samples are not available.

According to the 2017 data, 553,742 people, including 67% single adults and 33% individuals with families, were experiencing homelessness in the United States (Henry et al., 2017). Even though food insecurity is not universal among homeless people, they are more likely to experience food insecurity compared to their housed counterparts (D'Andreamatteo and Slater, 2018; Lee and Greif, 2008; Martin-Fernandez et al., 2018). Potential reasons for the higher food insecurity among homeless adults include the inability to purchase food, the inaccessibility of adequate cooking and food storage facilities, and being unable to access foods that meet dietary needs (Davis et al., 2008; Martin-Fernandez et al., 2018; Oliveira and Goldberg, 2002; Rodriguez et al., 2009).

Among domiciled samples, food insecurity has been associated with negative physical health outcomes, including obesity (Hernandez et al., 2017), hypertension (Venci and Lee, 2018; Wang et al., 2015) and diabetes (Venci and Lee, 2018; Wang et al., 2015). Food insecurity has also been associated with depression (Wang et al., 2015; Weiser et al., 2011; Whitaker et al., 2006) and post-traumatic stress disorder (PTSD) (Golin et al., 2016; Mugisha et al., 2015). While the research on food insecurity and health outcomes is scarce among homeless adults, several studies have found a positive association between food insecurity and mental health problems. For instance, food insecurity was positively associated with increased depressive symptoms among marginally-housed and homeless individuals living with HIV (Palar et al., 2015). However, the sample did not include unsheltered homeless individuals, among whom the severity of the symptoms and the odds of being depressed could be even higher. Among a sample of homeless women, Whitbeck et al. (2015) found food insecurity to be associated with PTSD. Among homeless adults, food insecurity has also been associated with psychiatric hospitalizations, higher rates of hospitalization for any cause, and more visits to the emergency department, compared with food secure, homeless individuals (Baggett et al., 2011).

Different explanations have emerged to account for the inequality of health outcomes among vulnerable populations including those experiencing homeless. Among them, the neomaterial perspective states that tangible factors, such as food and shelter, explain the diversity of health outcomes (Arcaya et al., 2015). Further poor health outcomes are the result of detrimental exposures and lack of material factors that arise as a consequence of unequal distribution of resources in a society (Lynch et al., 2000). Studies have shown that greater food insecurity is associated with poorer dietary intake, including macro and micro nutrition deficiencies (Lee and Frongillo, 2001; Weiser et al., 2011). Therefore, according to this theory poorer physical and mental health outcomes among food insecure individuals could be attributed to the lower quantity and/or quality of food consumed. Greater fruit and vegetable intake is an indicator of higher overall diet quality (Ramsay et al., 2017). Fruits and vegetables contain a range of nutrients and bioactive compounds such as vitamins, minerals, antioxidants, carotenoids, and flavonoids (Liu, 2013; Slavin and Lloyd, 2012), and numerous studies have shown greater daily fruit and vegetable intake to be associated with more desirable health outcomes (Boeing et al., 2012; Woodside et al., 2013).

On the other hand, the psychosocial theory states that the cause of poor health includes more than a lack of resources (i.e., food insecurity). Proponents of this perspective state that it is mainly the cascade of physiological responses that originate from perceived disadvantage in relationship to others, feelings of exclusion, stress and low social support that are at the origin of poor health outcomes (Arcaya et al., 2015; Lynch et al., 2000). According to this theory the association between food insecurity and poorer physical and mental health outcomes could be attributed to perceived disadvantages or an inability to cope with the external and internal stressors. The perceived or actual inability to handle aversive somatic or emotional states is defined as distress intolerance (McHugh et al., 2016). Studies have shown that lower distress tolerance could lead to adverse outcomes as individuals attempt to use maladaptive behaviors to cope with negative affect which could lead to poor physical or psychological outcomes (Simons and Gaher, 2005; Zvolensky et al., 2011). For example, Macatee et al. (2016) found lower distress tolerance to positively predict depressive symptoms and worry.

There is evidence of associations between food insecurity and poor health, depression, and PTSD among domiciled adults (Golin et al., 2016; Hernandez et al., 2017; Wang et al., 2015). However, there is scant evidence for such a relationship for adults experiencing homelessness even though they are disproportionately affected by food insecurity (Martin-Fernandez et al., 2018; Palar et al., 2015; Whitbeck et al., 2015). Further, there is no evidence of pathways that link the associations between food insecurity and poor health, depression, and PTSD among this vulnerable population. Based on the neomaterial perspective and psychosocial theory, there are two potential mediators of the association between food insecurity and poor health, depression and PTSD: 1) poorer diet quality (as measured by self-reported fruit and vegetable consumption in the current study) and 2) lower distress tolerance. The purpose of the current study is two-fold: 1) to examine the association between food insecurity and poor health, depression and PTSD; and 2) to investigate fruit/vegetable consumption and distress tolerance as potential links between food insecurity and the three health outcomes among adults experiencing homelessness.

## 2 Methods

### 2.1 Data and sample

There are 12 available emergency shelters in the Oklahoma City area serving about 1088 individuals experiencing homelessness according to 2016 Point In Time Count (Oklahoma City Planning Department, 2016). For the current study participants were recruited from six homeless shelters in Oklahoma City, OK during July–August 2016. Recruitment occurred through flyers posted at these shelters. Participants were eligible to enroll in the study if they were a minimum of 18 years of age, receiving shelter-based services (e.g., shelter, counseling) at the targeted shelters, and had a minimum 7th grade English literacy level based on a score of 4 or higher on the Rapid Estimate of Adult Literacy in Medicine-Short Form (REALM) (Arozullah et al., 2007). A total of 648 participants were screened for study participation and 38 were screened out due to lower literacy level. Specifically, 34 participants scored <4 on the REALM and an additional four participants could not read write or understand English. Eligible participants were provided with additional study details by research staff and allowed time to consider study enrollment. After providing informed consent, the potential participants completed questionnaires on a tablet computer. The questionnaire consists of 317 items and took about 63 min on average to complete the survey. Survey items were visible on the tablet screen and read aloud to the participant via headphones. A $20 department store gift card was provided to participants for their time. The Institutional Review Boards at the (omitted for review) and the (omitted for review) approved this study.

A total of 610 eligible adults enrolled in the study. Twenty-nine adults were excluded because they did not meet the criteria of being homeless (i.e. an individual that does not have a personal residence or other permanent location to sleep). Adults were further excluded from the analyses if they were missing data on the variables of interest: food security measure (4 excluded), health outcome data (2 excluded), or covariate data (9 excluded). The final analytic sample consisted of 566 homeless individuals.

### 2.2 Measures

#### 2.2.1 Food insecurity

Food security was measured using a six-item Food Security Scale-Short form developed by the National Center for Health Statistics in collaboration with Abt Associates Inc. (Blumberg et al., 1999). The scale assesses both the quality and quantity of a person's food over the past 12 months, along with whether they were able to afford the food they needed (e.g., “In the last 12 months, the food that you bought just didn't last, and you didn't have money to get more”). If participants responded affirmatively to zero or one of the six items, they were categorized as food secure. Individuals who responded affirmatively between two and six of the six items were categorized as being food insecure (Blumberg et al., 1999).

#### 2.2.2 Physical and mental health outcomes

Physical and mental health outcomes were assessed with three separate variables: Self-reported poor health status, depression, and post-traumatic stress disorder. Participants were asked to rate their overall health status on a 1 = *Excellent* to 5 = *Poor* scale. Those who rated their overall health status as “poor” or “fair” (4 or 5 on the scale) qualified as having poor health status (Businelle and Kendzor, 2016). Depression was assessed using the eight-item Patient Health Questionnaire (PHQ-8) (Spitzer et al., 1999). Participants who scored 10 points or higher were identified as experiencing depression. PTSD was assessed using the four item Primary Care Post-Traumatic Stress Disorder (PC-PTSD) screener. Those who responded yes to three or more items were described as experiencing PTSD symptoms (Prins et al., 2003).

#### 2.2.3 Mediators

Fruit and vegetable consumption and emotional distress tolerance variables were used as mediators. Fruit and vegetable consumption was analyzed as a continuous variable and was assessed through the question: “How many servings of fruits and vegetables did you consume on average each day during the past week? (A serving is ½ cup [4 ounces] of cooked vegetables, 1 cup [8 ounces] of salad, a piece of fruit, ¾ cup [6 ounces] of 100% fruit juice).” Emotional distress tolerance was assessed using the 16-item self-report Distress Tolerance Scale (DTS). This scale defines distress tolerance as one's ability to withstand emotional distress on a 5-point scale ranging from 1 = *Strongly Disagree* to 5 = *Strongly Agree*. Sample items include, “Feeling distressed or upset is unbearable to me”, “When I feel distressed or upset, all I can think about is how bad I feel” etc. The higher scores indicates lower levels of distress tolerance (Simons and Gaher, 2005).

#### 2.2.4 Covariates

A number of variables were included as covariates in the models that may influence mental and physical health and/or could be related to food security. Continuous covariates included age and the total number of years the individual has been homeless. The remaining covariates were all treated as dichotomous variables: sex (female or male), race/ethnicity (white/non-minority or minority), marital status (married or not married), education (less than a high school diploma or high school diploma or more), employment status (unemployed/disability limits employment or employed), sources of income (has no sources of income or has at least one source of income) and health insurance (insured to any extent or uninsured).

### 2.3 Analytic plan

Descriptive statistics on the study variables were conducted. Bivariate analyses comparing variables of interest by food security status were conducted using one-way analysis of variance tests for continuous variables and chi-square tests for dichotomous variables. Unadjusted logit models were conducted: 1) to evaluate the direct relationship between food insecurity and the three health outcome variables: poor health, depression and PTSD symptoms and 2) to assess the association between potential mediating variables to both exposure (food insecurity) and health outcome variables. For models that indicated significant paths for the two above assessments, direct and indirect relationships were examined through covariate-adjusted logit regression models. The indirect relationships were assessed using bootstrapping methods outlined by Preacher and Hayes (2004) with 5000 resamples. All analyses were performed using SPSS version 25 (Chicago, IL).

## 3 Results

The descriptive statistics for the study population are summarized in Table 1. Table 2 highlights the unadjusted logit regression models. The relationship between food insecurity and poor health (*b* = 0.90, *p* < .001), depression symptoms (*b* = 1.52, *p* < .001), and PTSD symptoms (*b* = 1.03, *p* < .001) was positive and significant (Panel A). Panel B indicates that food insecurity was not significantly related to fruit and vegetable consumption (*b* = −0.17, *p* = .504), and fruit and vegetable consumption was not significantly related to depression symptoms (*b* = −0.05, *p* = .189) or PTSD (*b* = −0.01, *p* = .736). Fruit and vegetable consumption was negatively and significantly related to poor health (*b* = −0.08, *p* < .05). Because fruit and vegetable consumption was not significantly associated with food insecurity (nor with depression and PTSD), covariate-adjusted mediation models were not applied.

**Table 1:** Descriptive statistics for study variables^a^ [M (SD) or %] (n = 566).

	Analytic sample (n = 566)	Food secure (n = 128)	Food insecure (n = 438)
Health outcomes			
Poor health	37%	22%	41%^⁎⁎⁎^
Depression	30%	11%	36%^⁎⁎⁎^
Post-traumatic stress disorder	32%	17%	37%^⁎⁎⁎^
Food security status			
Food insecure	77%	0%	100%
Potential mediators			
Food and vegetable consumption	3.9 (2.53)	4.05 (2.63)	3.88 (2.50)
Distress intolerance	2.90 (0.98)	2.47 (1.01)	3.02 (0.94)^⁎⁎⁎^
Control variables			
Age	43.50 (11.99)	43.49 (12.62)	43.51 (11.81)
Sex			
Female	36%	34%	37%
Male	64%	66%	63%
Race/ethnicity			
White/non-minority	57%	57%	56%
Minority	43%	43%	44%
Marital status			
Not married	88%	88%	88%
Married	12%	12%	12%
Education			
Less than high school diploma	26%	20%	28%
High school diploma or more	74%	80%	72%
Employment status			
Unemployed/disability limits employment	88%	91%	87%
Employed	12%	9%	13%
Sources of income			
No sources of income	54%	55%	53%
Has a source of income	46%	45%	47%
Health insurance			
No insurance	70%	73%	70%
Any insurance	30%	27%	30%
Number of years being homeless	3.19 (4.32)	2.80 (3.92)	3.30 (4.42)

^⁎^*p* < .05.

^⁎⁎^*p* < .01.

^⁎⁎⁎^*p* < .001.

a Oklahoma City, OK; July–August 2016; adults experiencing homelessness and accessing shelters. Distress Tolerance Scale was reverse coded so higher scores indicate greater distress intolerance.

**Table 2:** Unadjusted logit regression models of the direct relationship and association between potential mediating variables to both exposure (food insecurity) and outcome variables (health) (n = 566).

Panel A: Direct relationship			
	Health outcomes		
	Poor health	Depression	Post-traumatic stress disorder
Food insecurity	0.90 (0.23)^⁎⁎⁎^	1.52 (0.30)^⁎⁎⁎^	1.03 (0.25)^⁎⁎⁎^


Panel B: Potential mediator - fruit & vegetable consumption				
	Potential mediator	Health outcomes		
	Fruit & vegetable consumption	Poor health	Depression	Post-traumatic stress disorder
Food insecurity	−0.17 (0.25)	–	–	–
Fruit & vegetable consumption	–	−0.08 (0.04)^⁎^	−0.05 (0.04)	−0.01 (0.04)


Panel C: Potential mediator - distress intolerance				
	Potential mediator	Health outcomes		
	Distress intolerance	Poor health	Depression	Post-traumatic stress disorder
Food insecurity	−0.55 (0.10)^⁎⁎⁎^	–	–	–
Distress intolerance	–	0.54 (0.09)^⁎⁎⁎^	1.01 (0.11)^⁎⁎⁎^	0.72 (0.11)^⁎⁎⁎^

Note: Distress Tolerance Scale was reverse coded so higher scores indicate greater distress intolerance.

^⁎⁎⁎^*p* < .001.

^⁎⁎^*p* < .01.

^⁎^*p* < .05.

**Fig. 1:**
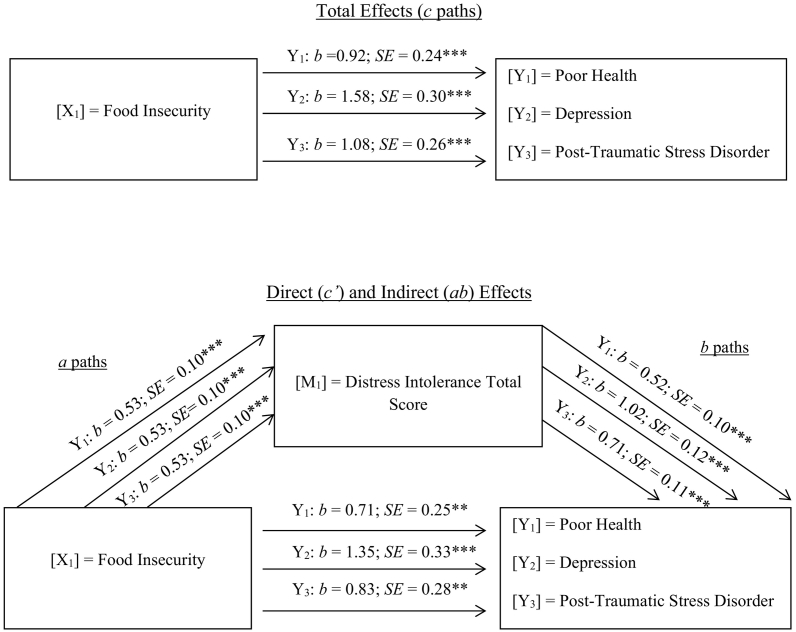
Covariate-adjusted model of the indirect effect (*ab*) of food insecurity on various physical and mental health outcomes through distress intolerance (n = 566). *Note*: ***p < .001, **p < .01, *p < .05. a path = Effect of X on M; b paths = Effect of M on Y; c paths = Total effect of X on Y; c′ paths = Direct effect of X on Y controlling for M. Three separate models were conducted. Distress Tolerance Scale was reverse coded so higher scores indicate greater distress intolerance.

The unadjusted models assessing emotional distress tolerance as an outcome of food insecurity and predictor of health measures were negative and significant (Table 2, Panel C). Therefore, covariate-adjusted mediation models were applied. According to covariate-adjusted models, the relationship between food insecurity and poor health (c path: *b* = 0.92, *p* < .001), depression symptoms (c path: *b* = 1.58, *p* < .001), and PTSD symptoms (c path: *b* = 1.08, *p* < .001) was positive and significant (Total Effects in Fig. 1). Next, emotional distress tolerance was examined as an indirect association between food insecurity and the three health outcomes. Food insecurity was positively and significantly related to distress tolerance (a path: *b* = 0.53, *p* < .001), and distress tolerance was positively and significantly related to poor health (b path: *b* = 0.52, *p* < .001), depression symptoms (b path: *b* = 1.02, *p* < .001), and PSTD symptoms (b path: *b* = 0.71, *p* < .001). The bootstrap tests of indirect effects indicated that lower levels of emotional distress tolerance partially mediated the association between food insecurity and poor health (*b* = 0.28, 95% CI: 0.14, 0.46), depression symptoms (*b* = 0.55, 95% CI: 0.32, 0.79), and PTSD symptoms (*b* = 0.38, 95% CI: 0.22, 0.57). The results support partial mediation because the total effect between food insecurity and the three health outcomes attenuated but was still significant after emotional distress tolerance was included in the models: poor health (c′ path: *b* = 0.71, *p* < .01), depression (c′ path: *b* = 1.35, *p* < .001), and PTSD symptoms (c′ path: *b* = 0.83, *p* < .01).

## 4 Discussion

In the current study, the majority of the participants were identified as experiencing food insecurity. This is expected based on the results of Gundersen et al. (2003), which reported that families with higher propensity towards homelessness have higher levels of food insecurity. Among those who were food insecure, a higher percentage of people reported poor health and experiencing depressive or PTSD symptoms. Our results are in line with past research done with food insecure adults from domiciled and homeless samples showing that they are more at risk for physical and mental health problems and more likely to rate their health as fair/poor (Decker and Flynn, 2018; Palar et al., 2015; Stuff et al., 2004).

Similar to previous research among domiciled samples (Boeing et al., 2012; Hosseini et al., 2017), fruit and vegetable consumption was inversely related to poor health status among homeless adults. However, in contrast to many previous study findings among the general population (Blanchflower et al., 2013; Liu et al., 2016; Wolniczak et al., 2017), we did not find a significant relation between fruit and vegetable consumption and mental health outcomes among homeless adults. Also, food insecurity was not related to the fruit and vegetable consumption. The fruit and vegetable consumption reported by homeless individuals in this study was higher than that reported by Americans adults in general (Lee-Kwan et al., 2017). These findings may be explained by homeless adults eating the food available to them at shelters which may include fruits and vegetables. This scenario would parallel research comparing fruit and vegetable consumption among food secure and food insecure youth in which children with food insecurity who participate in school meal programs consume greater amounts of fruits and vegetables than food secure youth (Grutzmacher and Gross, 2011; Widome et al., 2009).

In addition, our results did not support the hypothesis that fruit and vegetable intake would be a mediator linking food insecurity and poor health, depressive symptoms, and PTSD symptoms. Based on the neomaterial theory, the potential mediator is the dietary quality, and we used fruit and vegetable intake as a diet quality indicator. However, fruit and vegetable consumption is only one aspect of dietary quality. The use of a more comprehensive measure of dietary quality that includes multiple dietary components may have yielded different results. Further, the scale used to measure fruit and vegetable consumption is not based on an extensive 24-hour recall, which could provide greater insight into dietary intake compared to the current one-item question. Homeless individuals may have difficulty identifying constituents of their food as most of their food is from shelters and other sources where they have no involvement in food preparation. In addition, individuals tend to under/over report their dietary intakes which could have biased results (Subar et al., 2015). These study limitations are potential reasons for the non-significant results. More research is required to evaluate this relationship further using a more extensive measure of nutrient intake/diet quality.

In this sample, food insecurity was negatively related to emotional distress tolerance among homeless adults. Therefore, homeless adults experiencing food insecurity had lower scores for distress tolerance indicating that food deprivation or restriction is associated with lower ability to tolerate aversive somatic or emotional states. In addition, as reported in many empirical studies with domiciled samples, our study results show that distress tolerance is negatively associated with PTSD symptoms (Vujanovic et al., 2011, 2013) and depression symptoms (Magidson et al., 2013; Williams et al., 2013) among homeless adults. The results from the current study also support the hypothesis that distress tolerance functions as a partial mediator of the relationship between food insecurity and poor mental and physical health. In accordance with the psychosocial theory, food insecurity may be a trigger that lowers the ability to withstand emotional distress and thereby be a contributor to negative health outcomes.

Additional limitations not stated above include the cross-sectional design and the generalizability of the results. A longitudinal study may assist in better understanding how the temporal order and length of time associated with experiencing food insecurity and homelessness may contribute to emotional distress and negative physical and mental health outcomes. For example, there could also be a cumulative effect between the lifetime length of time of being food insecure and the lifetime length of time of being homeless that creates a high level of emotional distress that contributes to physical and mental health problems. It is also not clear from the data the length of time participants had been experiencing food insecurity within the 12 month boundary of the food insecurity measure, as well as beyond that period of time. Related, food security status could be measured with a more appropriate scale. The current overarching study questionnaire consisted of 317 items and an additional 26 screening items, resulting in total screening and survey participation to take over an hour. To reduce burden on the participants the Food Security Scale was used rather than the Household Food Insecurity Access Scale (HFIAS) which takes slightly longer to administer. However, future studies should consider the HFIAS, which is considered a better food security instrument to use among adults experiencing homelessness (Holland et al., 2011). Last, with the data collected from homeless adults currently receiving services at homeless shelters in Oklahoma City, OK, the findings cannot be generalized to all adults experiencing homelessness in other parts of the United States.

## 5 Conclusions

Our study suggests that among adults who experience homelessness there is a positive and significant relationship between food insecurity and poor health, depression symptoms, and PTSD symptoms. Further, low levels of distress tolerance among food insecure adults appear to be a contributing factor to poor physical and mental health status. It may be perceived that adults who experience homelessness and receive meals from shelters have less anxiety about their access to food and greater emotional tolerance. In the current study, 94% of the sample did receive meals from the shelter in the past 3 months; however this only occurred, on average 55 of the 90 days (results not shown). Thus, there may be confounding factors associated with food access that trigger emotional distress. Studies have shown that it is important to understand the specific food likes and dislikes of people in their shelters and try to provide nutritious, familiar and comforting food for these individuals (Truesdell and Sani, 2001). Culturally appropriate foods and the portability of those foods are important factors to consider when making food available to shelter-based adults. These factors may indirectly influence the physical and mental health of adults who experience homelessness.

## Funding

This research and preparation of this manuscript were supported by the Oklahoma Tobacco Settlement Endowment Trust (092-016-0002). Funding for this project was also supported by the American Cancer Society grant MRSGT-12-114-01-CPPB to the last author. The preparation of this manuscript was also partially supported by the National Cancer Institute 1P20CA221697-01 to the fifth author and subproject #5555 to the first author, and the Research and Extension Experiential Learning for Undergraduate (REEU) Program of the National Institute of Food and Agriculture, USDA, Grant # 2017-67032-26021 to the first and fourth author.
